# Specificity of the Human Frequency Following Response for Carrier and Modulation Frequency Assessed Using Adaptation

**DOI:** 10.1007/s10162-015-0533-9

**Published:** 2015-07-11

**Authors:** Hedwig E. Gockel, Alexandra Krugliak, Christopher J. Plack, Robert P. Carlyon

**Affiliations:** MRC-Cognition and Brain Sciences Unit, 15 Chaucer Road, Cambridge, CB2 7EF UK; School of Psychological Sciences, University of Manchester, Manchester Academic Health Science Centre, Manchester, M13 9PL UK

**Keywords:** human frequency following response, adaptation, phase locking, brainstem, selectivity

## Abstract

The frequency following response (FFR) is a scalp-recorded measure of phase-locked brainstem activity to stimulus-related periodicities. Three experiments investigated the specificity of the FFR for carrier and modulation frequency using adaptation. FFR waveforms evoked by alternating-polarity stimuli were averaged for each polarity and added, to enhance envelope, or subtracted, to enhance temporal fine structure information. The first experiment investigated peristimulus adaptation of the FFR for pure and complex tones as a function of stimulus frequency and fundamental frequency (F0). It showed more adaptation of the FFR in response to sounds with higher frequencies or F0s than to sounds with lower frequency or F0s. The second experiment investigated tuning to modulation rate in the FFR. The FFR to a complex tone with a modulation rate of 213 Hz was not reduced more by an adaptor that had the same modulation rate than by an adaptor with a different modulation rate (90 or 504 Hz), thus providing no evidence that the FFR originates mainly from neurons that respond selectively to the modulation rate of the stimulus. The third experiment investigated tuning to audio frequency in the FFR using pure tones. An adaptor that had the same frequency as the target (213 or 504 Hz) did not generally reduce the FFR to the target more than an adaptor that differed in frequency (by 1.24 octaves). Thus, there was no evidence that the FFR originated mainly from neurons tuned to the frequency of the target. Instead, the results are consistent with the suggestion that the FFR for low-frequency pure tones at medium to high levels mainly originates from neurons tuned to higher frequencies. Implications for the use and interpretation of the FFR are discussed.

## **INTRODUCTION**

An important obstacle to understanding the neural basis of human hearing is that, while a wide range of physiological techniques can be used with animals, many of those techniques are invasive and hence unsuitable for human participants. Although functional magnetic resonance imaging (fMRI) and magnetoencephalography can provide non-invasive and detailed measures of cortical activation, they are less good at revealing activity—with the desired high temporal resolution—in the many sub-cortical structures and pathways of the auditory system. In contrast, scalp-based electroencephalogram (EEG) techniques can measure subcortical processing with fine temporal precision, albeit at the expense of poor spatial resolution, and have the potential to provide useful insights into human hearing. The present article concerns one such measure, the frequency following response (FFR), which, using the electrode configuration employed here, is thought to reflect phase-locked activity at the level of the inferior colliculus (IC) and/or lateral lemniscus (LL). It has been proposed not only as a method for studying basic aspects of auditory function but also for assessing a range of disorders including reading problems in children (Hornickel et al. 2012) and “hidden hearing loss,” i.e., auditory neuropathy that is not thought to be detectable by measuring standard audiometric thresholds (Bharadwaj et al. 2014).

Despite the widespread use of the FFR in scientific and clinical studies, the extent to which it represents auditory processes that are important for human hearing has been questioned on at least two grounds. First, it has been argued that the FFR to low-frequency pure tones at medium-to-high-levels is generated not from neurons tuned to the tone frequency, but from neurons having a wide range of characteristic frequencies (CFs) above the tone frequency (Dau 2003). In realistic listening environments, where more than one frequency component is present, it is probable that the frequency of each component is encoded by the temporal response of neurons tuned to that component (Young and Sachs 1979; Young 2008). In addition, evidence from listeners with “dead” regions of localized hair cell loss suggests that, when the temporal information is conveyed by neurons with CFs remote from the signal frequency, the pitch percept is weak and degraded (Huss and Moore 2005a, b). Thus, if the FFR originates mainly from off-frequency neurons, it may only poorly reflect the neural mechanisms underlying the pitch and perceived quality of the sounds. Second, recent evidence from our laboratory has questioned the idea that the FFR reflects pitch processing as often assumed (Gockel et al. 2011). That evidence came from the finding that the spectrum of the FFR to a dichotic harmonic complex, in which different components are presented to opposite ears, does not correspond to the perceived pitch but, instead, resembles the sum of the responses to each ear’s input presented separately.

The present experiments use an adaptation paradigm to gain further insight into the neural basis of the FFR, to compare FFR adaptation to that observed in single- and multi-unit recordings from animals, and to seek evidence for frequency selectivity in the modulation domain. The first experiment studied the extent to which neural adaptation is reflected in the FFR and measured the dependence of peristimulus adaptation on the frequency of a pure tone and on the fundamental frequency (F0) of a complex tone consisting of low-numbered, resolved harmonics. There was more adaptation for sounds with a higher frequency or F0 than for sounds with a lower frequency or F0.

To control for the effects of frequency region, experiment 2 measured adaptation to complex tones containing only high-numbered peripherally unresolved harmonics, bandpass filtered into a fixed frequency region, and with a pitch corresponding to the modulation rate of the envelope. Peristimulus adaptation was greater when the modulation rate was 504 Hz than when it was 213 Hz, indicating an effect on adaptation of modulation rate per se, independently of frequency region—and hence, by implication—of the CFs of the neurons whose response we measured.

Another major aim of experiment 2 was to determine whether the FFR could provide evidence for, and an objective measure of, modulation specificity in neurons in the upper brainstem. To do so, adaptation of the FFR was measured in a “forward masking” paradigm; that is, we measured the response to the start of a “probe” unresolved complex tone preceded by an adapting complex tone that could have either the same or a different modulation rate. The probe FFR was not reduced more when the adaptor had the same than when it had a different modulation rate, thus providing no evidence for modulation rate selectivity in the FFR.

Experiment 3 studied audio-frequency selectivity using pure tone adaptors and probes with all possible combinations of two frequencies (213 and 504 Hz). Adaptation of the probe was always greater for the higher-frequency adaptor, regardless of the probe frequency. Hence, over this range of frequencies, and for sounds of moderate loudness, there was little or no frequency selectivity in FFR adaptation, supporting the idea that the FFR to low-frequency tones of moderate-to-high levels reflects the response of neurons tuned to a wide range of frequencies.

## **EXPERIMENT 1: EFFECT OF FREQUENCY And f0 ON ADAPTATION**

### Rationale

The FFR is well known to show a low-pass behavior, i.e., the strength of the FFR is greater at lower than at higher stimulus-related frequencies (see, e.g., Gockel et al. 2011; Zhu et al. 2013). Experiment 1 aimed to determine whether adaptation of the human FFR would also depend on stimulus frequency and/or F0. Some suggestion that this could be the case comes from animal physiology. Shackleton et al. (2009) recorded responses of multi-unit clusters in the central nucleus of the IC of anesthetized guinea pigs to wideband harmonic complex tones of various F0s. For all clusters, they observed sustained responses (firing rates) at low F0s but more adaptation for higher F0s.

### Methods

#### Stimuli

The FFR was recorded for pure tones and for harmonic complex tones. Pure tone frequencies were 244 and 504 Hz. Complex tones comprised harmonics three to five for F0s of 244 or 504 Hz, with starting phases of the components of 0°, 120°, and 240° for the 3rd, 4th, and 5th harmonics, respectively. Complex tones were presented at a root-mean-square (rms) level of 75 dB SPL. Pure tones were presented at three levels, spaced by 11-dB intervals. To equate sensation levels across frequency, the level of the 244-Hz sinusoid was 6 dB higher than that of the corresponding 504-Hz sinusoid (ISO 389–7 2005; Moore 2012). Presentation levels were 54, 65, and 76 dB SPL for the 244-Hz sinusoid and 48, 59, and 70 dB SPL for the 504-Hz sinusoid. Stimulus duration was 100 ms, including 5-ms raised-cosine rise/fall times.

Stimuli were generated with 16-bit resolution and a sampling rate of 40 kHz. They were played out through the digital-to-analog converter included in the evoked potentials acquisition system (Intelligent Hearing Systems-Smart-EP, IHS) and presented binaurally through mu-metal shielded Etymotic Research ER2 insert earphones, which have a flat frequency response at the human eardrum.

#### Subjects

Ten subjects (seven females) participated. They ranged in age between 18 and 35 years. All had self-reported normal hearing, and for both ears, pure tone thresholds were below 20 dB HL at octave frequencies from 250 to 4000 Hz. Six of them had some musical training. Informed consent was obtained from all subjects in this and all following experiments. This study was carried out in accordance with the UK regulations governing biomedical research and was approved by the Cambridge Psychology Research Ethics Committee.

#### Electrophysiological Recording

Subjects laid comfortably (in a reclining chair) in a double-walled electrically shielded sound-attenuating booth. They were instructed to relax and to refrain as much as possible from moving during sound presentation and recording. They were allowed to fall asleep. The FFR was recorded differentially between gold-plated scalp electrodes positioned at the midline of the forehead at the hairline (+, Fz) and at the seventh cervical vertebra (−, C7). A third electrode placed on the mid-forehead (Fpz) served as the common ground. For this “vertical” electrode montage, the FFR is generally assumed to reflect sustained phase-locked neural activity mainly from rostral generators of the brainstem (IC and lateral lemniscus, LL, Marsh et al. 1975; Smith et al. 1975; Glaser et al. 1976; Galbraith 1994; Krishnan 2006). Electrode impedances were less than 1 kΩ for all recordings. The FFR signal was recorded with a sampling period of 0.075 ms, amplified by a factor of 100,000 and bandpass filtered from 50 to 3000 Hz (6 dB/octave roll-off, resistor-capacitor filter). Epochs with electric potential changes exceeding 31 μV were automatically discarded and the trial repeated. The polarity of the stimuli was alternated for each presentation, and alternate-polarity sweeps were recorded and averaged in separate data buffers by the SmartEP system. Stimuli were played with a repetition rate of 3.57/s, corresponding to a repetition period of 280.11 ms. The same stimulus was played in blocks of 3072 (valid) trials. The order of conditions was randomized across subjects, with the restriction that the two complex-tone conditions were blocked at either the beginning or the end of the session (for half of the subjects each). Data were collected in a single session of about 3 h, including electrode placement and breaks. Control recordings in which all of the same procedures were followed, but with the tubes of the insert earphones blocked, resulted in no signal above the noise floor at stimulus component, envelope, or distortion product frequencies in the subtraction waveform (see below) of the FFR.

#### Analysis

Offline processing was done using MATLAB (The Mathworks, Natick, MA). In this and all following experiments, first, for complex tones, the averaged FFR responses for original-polarity and for inverted-polarity stimuli were added, while for pure tones they were subtracted from each other, and the result divided by two for each subject and condition. Addition of responses to alternating-polarity stimuli enhances the representation of phase-locked activity to the envelope of the stimulus and minimizes the representation of phase locking in response to the temporal fine structure. Subtraction of responses to alternating-polarity stimuli enhances the representation of phase-locked activity in response to the temporal fine structure and minimizes the representation of phase locking to the envelope of the stimulus (Goblick and Pfeiffer 1969; Aiken and Picton 2008). All further analyses are based on these averaged addition waveforms (for complex tones) and subtraction waveforms (for pure tones). The FFR was analyzed and compared across three 30-ms time ranges: (i) from 30 ms before stimulus onset to stimulus onset (baseline), (ii) from 12 to 42 ms after stimulus onset (start; the value of 12 ms was chosen such that the beginning of the FFR onset response was excluded and is the same as usually used in our laboratory (Gockel et al. 2011; 2012; 2013)), and (iii) from 70 to 100 ms after stimulus onset (end).

For spectral analysis, for each subject, the 30-ms waveform was zero-padded symmetrically to make up a 1-s signal, and the magnitude spectrum was calculated via a discrete Fourier transform. The dependent measure for the amount of phase-locked neural activity (the FFR strength) was defined as the highest magnitude present in the spectrum within a 34-Hz range centered on the frequency of the pure tone or the envelope rate for the complex tone. The magnitude spectrum is specified in decibels re 0.01 μV. We assume that adaptation is appropriately quantified as the ratio of FFR magnitudes before and after adaptation, rather than as the difference in magnitudes. Hence, we use a dB scale throughout, where measured changes are not susceptible to, e.g., the distance of the electrodes from the source; see McKay (2012) for clear arguments on this point.

The data for the lowest level pure tone conditions (54 and 48 dB SPL for the 244-Hz and the 504-Hz pure tones, respectively) are not reported here, as most subjects did not show a clear FFR in these conditions. The data from two subjects were excluded from further analysis. Their FFR magnitude at time start was less than 5 dB above the baseline for at least one of the higher level conditions and was not above baseline (criterion for a response to be considered present) in at least one of the medium-level pure tone conditions.

Statistical analysis (repeated-measures analyses of variance, RM ANOVAs, and *t* tests) was performed on the spectral magnitudes at the frequencies of interest (expressed in dB) using SPSS (Chicago, IL). Throughout the paper, if appropriate, the Huynh-Feldt correction was applied to the degrees of freedom for the ANOVAs (Howell 1997). In such cases, the original degrees of freedom and the corrected significance value are reported. Throughout the paper, *t* tests were two-sided, and the Bonferroni correction was applied to the *p* values of multiple *t* tests following a significant ANOVA. To reflect the within-subject design used in all experiments, the error bars in all figures show the standard error of the mean (sem) based on normalized data—to equate the mean performance across subjects—as intersubject variance is irrelevant for within-subject design (Loftus and Masson 1994). Specifically, for each subject S, the FFR strength (in dB) for each condition was normalized by: (1) adding the mean FFR strength averaged across all subjects and conditions and (2) subtracting the mean FFR strength averaged across all conditions for subject S.

### Results

**FIG. 1:**
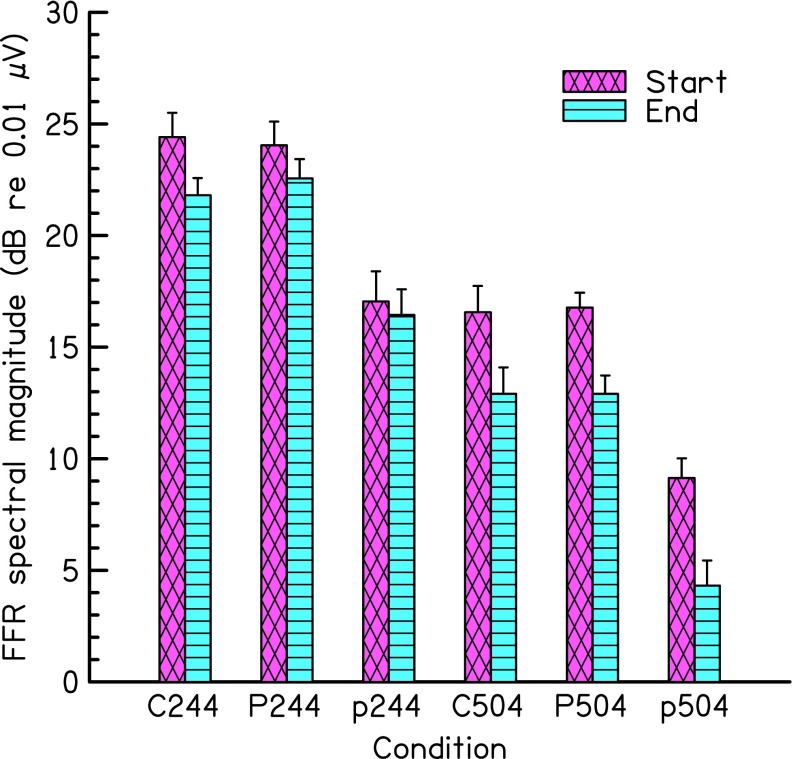
Results of experiment 1. Mean FFR spectral magnitude at the frequency corresponding to the envelope rate or frequency of the stimulus and the corresponding standard errors across 8 subjects. Each group of two bars shows the FFR magnitude during two time ranges of the stimulus: 12–42 ms after onset (start) and 70–100 ms after onset (end). Conditions: C244 and C504 = 75-dB SPL complex tones, containing harmonics 3–5 of 244- and 504-Hz F0s, respectively; P244 and p244 = 244-Hz pure tones at 76 and 65 dB SPL, respectively; P504 and p504 = 504-Hz pure tones at 70 and 59 dB SPL, respectively. The error bars in this and the following figures show the standard error of the mean based on normalized data—to equate the mean performance across subjects—as intersubject variance is irrelevant for within-subject design (see Methods).

Figure 1 shows the FFR strength for the complex tones (C244 and C504) and for the pure tones for the high (P244 and P504) and medium (p244 and p504) levels, averaged across subjects, and the corresponding standard error across subjects. As expected, the FFR was generally larger for the stimuli with a repetition rate of 244 Hz (three groups of bars on the left-hand side) than for the corresponding stimuli with a 504-Hz repetition rate (three groups of bars on the right-hand side). In addition, for the pure tones, the FFR was larger at the high than at the medium level, as expected. Some adaptation over time (reduction in FFR strength from the start to the end time ranges) occurred for all conditions, and there was more adaptation for the higher rate than for the lower rate stimuli.

A three-way RM ANOVA (with factors (i) repetition rate: 244 vs 504 Hz, (ii) time: start vs end, and (iii) stimulus type: complex tone vs pure tone) was calculated on the FFR measures for the complex and the high level pure tones. The main effects of repetition rate and time were both highly significant [F (1,7) = 80.36, *p* < 0.001, and F (1,7) = 28.13, *p* = 0.001, respectively], while the effect of stimulus type was not (*p* = 0.92). Importantly, the interaction between repetition rate and time was significant [F (1,7) = 10.19, *p* = 0.015], indicating that there was significantly more adaptation over time for the 504-Hz than for the 244-Hz rate stimuli. No other interaction was significant. A second RM ANOVA was calculated on the FFR measures for the pure tones only (with factors frequency, time, and stimulus level). All three main effects were significant [frequency: F (1,7) = 43.34, *p* < 0.001; time: F(1,7) = 9.63, *p* = 0.017; level: F (1,7) = 107.16, *p* < 0.001]. Again, the interaction between frequency and time was significant [F (1,7) = 12.19, *p* = 0.010], indicating that adaptation was significantly stronger for the 504-Hz than for the 244-Hz tones. No other interaction was significant. Theoretically, the stronger adaptation for the 504-Hz tones than for the 213-Hz tones could be a consequence of the weaker FFR for the 504-Hz than for the 213-Hz tones. To test this, an additional RM ANOVA was calculated on the FFR strength for p244 and P504, for which the non-adapted FFR was similar. As in the previous ANOVAs (where the FFR for the 504-Hz tones was weaker than that for the 213-Hz tones), the interaction between stimulus frequency and time was significant [F (1,7) = 13.86, *p* = 0.007], and thus, the larger adaptation for the higher-frequency tone did not depend on a difference in overall FFR strength.

The results showed that adaptation of the human FFR was larger for higher-rate than for lower-rate stimuli, both for pure tones and for complex tones. For the complex tones, this effect could have been mediated by the component frequencies and/or the envelope rate, as the complex tones differed in both.

### Discussion

We consider first the extent to which our results resemble those found in neurophysiological studies in animals. All of these studies measured adaptation of the firing rate (not of the synchronization index) of either individual neurons or multi-unit clusters in anesthetized animals. Of course, the FFR reflects sustained phase locked activity in large populations of neurons, and thus, as the relative timing of the firing of neurons is crucial, it cannot be predicted solely on the basis of firing rates of individual neurons. Nevertheless, all other things being equal, adaptation of spike rate in single cells or multi-unit clusters may affect the FFR measured from the human scalp.

In the gerbil, it has been shown that the time course of spike rate adaptation of auditory nerve fibers (ANFs) during pure tone bursts is related to neural characteristic frequency (CF); ANFs with higher CFs tend to adapt more quickly than ANFs with lower CFs (Westerman and Smith 1984, 1985; Crumling and Saunders 2007). Westerman and Smith (1984) measured peristimulus adaptation in single ANFs of the Mongolian gerbil using tones at the CF of the fiber. They showed that the time constant for the initial rapid part of the adaptation was shorter for high-CF fibers than for low-CF fibers (for tones presented at 40–43 dB above unit threshold) and tended to decrease with increasing level. In a similar experiment, Westerman and Smith (1985) presented tones at the CF and tones one octave below the CF of the fiber, each at 20 dB above the fiber’s threshold for that frequency. Again, they found that, for stimulation at CF, the higher-CF fibers adapted more quickly than lower-CF fibers. However, lowering the stimulus frequency for a given fiber mostly did not decrease the rate of adaptation. Thus, they did not find an effect of stimulus frequency per se. Crumling and Saunders (2007) measured peristimulus adaptation in single units of the chick AN using 100-ms pure tone bursts at CF at 20 dB above the unit threshold. They reported a progressive increase in the amount of adaptation with increasing CF. They also used tone bursts below and above CF, with stimulus levels fixed at 20 dB above the unit threshold at that frequency and showed that the faster adaptation at higher CFs appears to be partly a consequence of the dependence of adaptation rate on the tone frequency as such, due to more rapid transmitter depletion at higher stimulating frequencies.

At the level of the IC, Dean et al. (2008) showed that firing rates of single units in the guinea pig adapted more quickly following an increase in stimulus level for high-CF neurons than for low-CF neurons. The total adaptation was greater for rapidly adapting neurons than for slowly adapting neurons. Furthermore, as discussed above, Shackleton et al. (2009) reported more adaptation for higher-than for lower-F0 complex tones in the firing rate of all multi-unit clusters in the central nucleus of the IC of guinea pigs, with the CFs of the clusters varying over a wide range. In summary, animal experiments have shown that spike-rate adaptation in both the AN and IC depends not only on CF, but also to some extent on stimulus frequency (Crumling and Saunders 2007; Shackleton et al. 2009).

As mentioned above, the FFR cannot be predicted solely on the basis of firing rates of individual neurons as it reflects phase-locked activity in large populations of neurons for which the relative timing of the responses is crucial. Nevertheless, the present new finding that there is more adaptation for higher than for lower frequencies and F0s of the FFR in humans is largely in agreement with results based on single-unit and multi-unit cluster recordings in animals. Factors contributing to this greater adaptation could be different neural populations—with different CFs and different adaptation characteristics—and/or the different stimulus frequencies and F0s per se. Such differences in amounts of adaptation might affect comparisons between FFR measures for stimuli of different frequencies and duration.

## **EXPERIMENT 2: SPECIFICITY OF ffr FOR MODULATION RATE**

### Rationale

A major aim of experiment 2 was to determine whether the FFR could provide evidence for, and an objective measure of, modulation specificity in neurons in the upper brainstem. Psychophysical evidence for selectivity in the modulation domain initially came from experiments where modulation detection thresholds for a target modulation were measured in the presence of simultaneous maskers with various modulation rates. Typically, maskers were most effective when the modulation rate of the masker was similar to that of the target (see, e.g., Bacon and Grantham 1989; Houtgast 1989). These modulation-masking data have inspired models of envelope processing based on banks of filters selective for modulation frequency for each peripheral auditory channel (Dau et al. 1997). Psychophysical evidence for modulation-rate selectivity in the modulation domain has also come from experiments investigating long-term adaptation (Richards et al. 1997; Wojtczak and Viemeister 2003) and forward masking (Wojtczak and Viemeister 2005; Moore et al. 2009) in the modulation domain. In addition, physiological experiments have revealed neurons in the IC (Langner and Schreiner 1988; Joris et al. 2004) and in the ventral nucleus of the LL (Batra 2006; Zhang and Kelly 2006; Recio-Spinoso and Joris 2014) that are tuned to different AM frequencies. As mentioned above, the FFR is thought to reflect phase-locked activity at the level of the IC and/or the LL. The best AM frequencies (the AM frequencies that maximize either the synchrony of firing or the firing rates) of neurons tuned to AM depend on species and the recording site. For example, in the IC of the unanesthetized rabbit, the best AM frequencies in terms of synchronized rate (the product of synchronization coefficient and average firing rate) ranged from 11 Hz (mean minus SD) to 193 Hz (mean plus SD) (Batra et al. 1989). In contrast, in the ventral nucleus of the LL of anesthetized rats, best AM frequencies in terms of firing rate ranged from 10 to 300 Hz or from 10 to 400 Hz, depending on the type of neuron, and ranged from 10 to 400 Hz in terms of vector strength (a measure of synchronization) (Zhang and Kelly 2006). In the ventral nucleus of the LL of anesthetized cats, the best AM frequencies in terms of firing rate ranged even up to 900 Hz, with strong synchronization to the envelope waveform over the entire range of responsiveness (Recio-Spinoso and Joris 2014). Furthermore, an fMRI experiment with macaques has provided evidence that modulation rate is represented at the level of the IC along a dimension that is orthogonal to that for CF (Baumann et al. 2011). That study used broadband noise with modulation rates ranging from 0.5 to 512 Hz, thus including rates high enough to evoke a musical pitch.

Here, we investigated tuning to modulation rate in the FFR using adaptation. The experiment employed complex tones with unresolved harmonics filtered into the same frequency region, allowing the effects of modulation rate to be evaluated without a corresponding and potentially confounding co-variation in center frequency. The rationale in this and the following experiments is based on the assumption that an adaptor will reduce the response to a target more when it adapts neurons responding most strongly to the target than when it adapts neurons that respond less strongly to the target (Näätänen et al. 1988). The experimental question was whether the FFR evoked by a target sound with a given modulation rate would be reduced more by an adaptor that had the same modulation rate than by adaptors with different modulation rates. If such specificity for modulation rate of the adaptor were observed in the FFR of the target, this would provide evidence that the FFR originates mainly from neurons that respond selectively to the modulation rate of the stimulus and would provide physiological evidence for the existence of filters tuned to modulation rates in humans. In addition, experiment 2 helped to control for the effects of CF on the amount of peristimulus adaptation as, in contrast to experiment 1, the frequency region of the various complex tones (used as adaptors) was kept roughly constant.

### Methods

The FFR evoked by a 100-ms, 75-dB SPL complex-tone target with 213 envelope peaks per second (defined as envelope rate) was measured. The target was always preceded by a 200-ms adaptor and followed the adaptor without any silent gap. The different adaptor conditions are specified below. All tone durations included 5-ms raised-cosine rise/fall times. All complex tones were composed of alternating-phase harmonics (even harmonics added in cosine and odd harmonics added in sine phase). They were filtered into the frequency range between 3.9 and 5.4 kHz, which was high enough to ensure that the harmonics were not resolved and interacted strongly in the peripheral auditory system. Under these conditions, the number of envelope peaks per second is twice the F0. This leads to a pitch corresponding to twice the F0 (Shackleton and Carlyon 1994), and major spectral peaks in the FFR addition waveform at the envelope rate (corresponding to the pitch of the complex) and integer multiples thereof (Krishnan and Plack 2011). Filtering of the complex tones into a high-frequency region and alternating the starting phases of harmonics allowed us to employ complex tones with a pitch in the musical range, and within the range used by Baumann et al. (2011), without the presence of resolved harmonics.

When the adaptor and target had the same envelope rate, the target component phases were “preserved” relative to those for the adaptor, i.e., the phase of each component in the target was the same as if the adaptor had continued; the adaptor and target comprised the first 200 and the last 100 ms of a 300-ms complex, respectively, with onset and offset ramps applied independently on each. The starting phases of components in the target were identical in all conditions. When the adaptor envelope rate differed from that of the target, a temporal offset was applied to the adaptor so that its last major envelope peak occurred at the same time as for the 213-Hz rate adaptor.

Stimulus generation and presentation and analysis of the recorded signal were done in the same way as in experiment 1, with the following exceptions: stimuli were played with a repetition rate of 1.81/s. The same stimulus condition was played in blocks of 1500 (valid) trials; two blocks were run for each condition in randomized order across subjects, giving 3000 valid trials per condition. The FFR was recorded with a sampling rate of 10 kHz. The FFR was analyzed and compared across five 50-ms time ranges: (1) from 15 to 65 ms after adaptor onset (A-start; the value of the starting time was increased from 12 ms, as used in experiment 1, to 15 ms in experiment 2A because of the later response onset of the FFR for the adaptor with the lowest envelope rate (see below) for which the first pulse in the physical stimulus appears later than for higher rate stimuli); (2) from 65 to 115 ms after adaptor onset (A-mid); (3) from 150 to 200 ms after adaptor onset (A-end); (4) from 12 to 62 ms after target onset (T-start); (5) from 50 to 100 ms after target onset (T-end). The FFR for the 50-ms window before adaptor onset served as baseline.

For spectral analysis, for each subject, the 50-ms waveform was zero-padded symmetrically to make up a 1-s signal, and the magnitude spectrum was calculated via a discrete Fourier transform. The magnitude spectrum is specified in decibels re 0.01 μV. The FFR strength was defined as the highest magnitude present in the spectrum within a 20-Hz range centered at the envelope rate of the signal, i.e., at 213 Hz for the target and at the envelope rate of the adaptor when the adaptor was a complex tone, or the audio frequency of the adaptor when it was a pure tone.

### Experiment 2A: Adaptor at 75 dB SPL

#### Stimuli

The 213-Hz complex-tone target was always preceded by a 200-ms, 75-dB SPL adaptor, i.e., adaptor and target had the same rms level. There were four conditions. In three of these, the adaptor was a complex tone with an envelope rate of 90, 213, or 504 Hz (conditions AC90, AC213, and AC504, respectively). Thus, the envelope rate of the adaptor was either identical to that of the target (AC213) or differed by 1.24 octaves. In psychophysical experiments, detection thresholds for modulation of a probe that followed a modulated adaptor clearly differed when the adaptor’s modulation rate was identical to that of the probe compared to when it was changed from that of the probe by 1.24 octaves (Wojtczak and Viemeister 2005; Moore et al. 2009). If the FFR reflects behavioral performance, one might expect to observe some differences in the FFR strength for the target (T-start). In the fourth condition (AP213), the adaptor was a 213-Hz pure tone, i.e., a tone corresponding in frequency to a distortion component at the envelope repetition rate. For brevity, we will refer to this distortion component as “QDP,” as a quadratic nonlinearity would produce a distortion component at this frequency (see, e.g., Pressnitzer and Patterson 2001). Condition AP213 was included in order to check that the FFR we measured, and any adaptation of it, was not determined by the QDP that propagated from its place of generation in the cochlea to its characteristic place. If it were, then any specificity to modulation rate that we observed might instead be due to specificity to the audio frequency of the QDP. Experiments measuring the FFR to the modulation rate of unresolved complexes typically use noise to mask the QDP (Wile and Balaban 2007; Carcagno and Plack 2011; Krishnan and Plack 2011). However, as we have argued elsewhere (Gockel et al. 2012), this noise is not always effective and can potentially affect the FFR in other ways, such as via wideband inhibition. By including an adaptor at the frequency of the QDP, and comparing its effects to that of a complex tone adaptor filtered into the same frequency region as the probe, we could provide an alternative way of checking whether the FFR was affected more by the QDP or by the envelope of the complex tone.

#### Subjects

Ten subjects (four male) participated. They ranged in age from 19 to 31 years and had self-reported normal hearing. The ten were selected from a pool of 14 subjects because they showed clear FFR signals to the target alone and to the adaptors; at time A-start, their FFR was at least 10 dB and on average 17 dB above baseline for condition AC213 and at least 6 dB (average 15 dB) above baseline for all other conditions. Five of them had some musical training. The experiment lasted about 3 h, including breaks.

#### Results

**FIG. 2:**
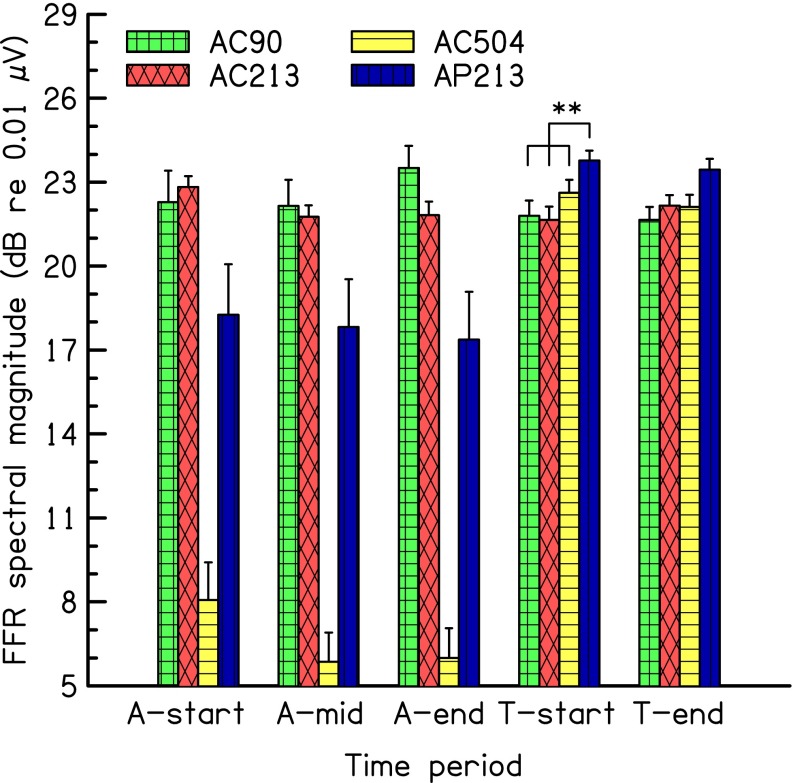
Results of experiment 2A. Mean FFR spectral magnitude at the frequency corresponding to the envelope rate or frequency, for the pure tone adaptor only, of the stimulus (adaptor or target) and the corresponding standard errors across 10 subjects. The three left-hand groups of four bars show the FFR strength during three time ranges of stimulation with the adaptor: 15–65 ms after onset (A-start), 65–115 ms after onset (A-mid), and 150–200 ms after onset (A-end). The two right-hand groups of four bars show the FFR strength during two time ranges of stimulation with the target: 12–62 ms after onset (T-start), 50–100 ms after onset (T-end). The bar patterns and colors code the types of adaptor that precede the 213-Hz envelope rate complex-tone target (see legend). Adaptor and target at 75 dB SPL; **p < 0.01.

Figure 2 shows the FFR strength averaged across subjects and the corresponding standard error across subjects. Adaptation over time occurred in the FFR in response to all adaptors (three groups of bars on the left-hand side), except for the 90-Hz envelope rate (green bars) where the FFR increased over the time course of the adaptor.

An RM two-way ANOVA (with factors time, A-start vs A-end, and four adaptor types) was calculated on the FFR for all adaptors. The main effect of adaptor type was highly significant [F (3,27) = 30.41, *p* < 0.001], while the effect of time was not (*p* = 0.16), but there was a significant interaction between type of adaptor and time [F (3,27) = 5.27, *p* = 0.011]. The interaction was driven by the 90-Hz envelope rate adaptor for which the FFR *increased* rather than decreased over time. A second ANOVA, without the 90-Hz envelope rate adaptor, showed that the FFR strength was significantly larger during period A-start than during period A-end [F (1,9) = 5.84, *p* = 0.039]. The main effect of adaptor type was highly significant [F (2,18) = 33.97, *p* < 0.001], and there was no significant interaction between type of adaptor and time (*p* = 0.33, after the exclusion of the 90-Hz adaptor). Note that excluding the 504-Hz rather than the 90-Hz adaptor from the ANOVA did not eliminate the significant interaction between type of adaptor and time [F (2,18) = 6.24, *p* = 0.009], with no main effect being significant, showing that the pattern over time observed for the 90-Hz adaptor differed from that observed for the other adaptors. Individual *t* tests, calculated for each adaptor type separately, showed that the FFR strength was significantly larger during period A-start than during period A-end for condition AC213 [difference = 1.0 dB, t (9) = 3.63, *p* = 0.022], but missed significance for condition AC504 and AP213 [difference = 2.1 dB, *p* = 0.296 and difference = 0.9 dB, *p* = 0.967, respectively] and was non-significantly *smaller* during period A-start than during period A-end for condition AC90 [difference = −1.2, t (9) = −2.43, *p* = 0.152]. For condition AC213, the FFR strength was significantly larger during period A-start than during period T-start [difference = 1.2 dB, t (9) = 2.95, *p* = 0.016], showing that the presence of the short offset and onset ramps in this otherwise “continuous” complex did not lead to complete recovery from adaptation for the target.

The observed increase in FFR over the time course of the 90-Hz envelope rate adaptor (which led to a significant interaction between type of adaptor and time) was unexpected, and we do not have a definite explanation for it. One possible explanation is that, for the 90-Hz adaptor, two generators contributed to the FFR; if the two generators were out of phase, then adaptation of one of them could lead to an increase in the FFR. Another possible explanation is connected with the small number of envelope periods within the analysis time window for the 90-Hz rate adaptor. Reducing the duration of the analysis time window (from 50 to 21.13 ms) for the 213-Hz rate adaptors (conditions AC213 and AP213), so as to contain the same number of cycles as for the 90-Hz rate in the 50-ms windows, did not generally result in an increase of the FFR from the first to the fourth time windows. However, it did introduce non-monotonic FFR strengths over the first four time windows for both 213-Hz rate adaptors, indicating that increased measurement variability might contribute to the observed increase in the FFR for the 90-Hz adaptor.

Any effects of the adaptor on the target FFR were expected to be strongest at the beginning of the target. For this reason, in this and the following experiments, the statistical analyses were restricted to T-start, but the figures include T-end for completeness. To assess whether there was any evidence for envelope-rate-selective adaptation in the FFR for the target, an RM one-way ANOVA was calculated on the FFR strength for the target during period T-start (Fig. 2, fourth cluster of bars from the left) for all conditions with a complex tone adaptor. The effect of adaptor envelope rate was not significant (*p* = 0.12). Thus, there is no evidence that the complex tone adaptor with an envelope rate equal to that of the target led to more adaptation of the target FFR than the complex tone adaptors with envelope rates different from that of the target.

To compare the FFR strength at time T-start across *all* adaptor conditions, i.e., the complex tone adaptors *and* the pure tone adaptor which occupied a different frequency region, an RM one-way ANOVA was calculated on the FFR during period T-start for all adaptors. This showed that the FFR strength differed significantly across adaptor conditions [F (3,27) = 9.27, *p* < 0.001]. To assess whether there was any evidence for carrier-frequency-selective adaptation, the FFR strength during period T-start was compared between the pure tone and the complex tone adaptor conditions. Individual *t* tests showed that the FFR strength during period T-start was significantly larger after the pure tone adaptor (AP213) than after any of the complex tone adaptors [p = 0.003, *p* = 0.009, and *p* = 0.006 for the comparison of AP213 with AC213, AC90 and AC504, respectively]. Thus, there was a significant effect of frequency region (or/and the absence or presence of modulation), with more adaptation when the adaptor was in the same frequency region as the target (or/and was modulated) than when it was below the target. After the pure tone adaptor, the target FFR was slightly greater than at the beginning of the AC213 adaptor [difference = 1 dB, t (9) = 2.34, *p* = 0.044]. This could be the consequence of accumulated adaptation across trials, and will be discussed below.

The lack of adaptation of the target FFR in condition AP213 also indicates that the FFR to the target is not mainly driven by neurons with CFs corresponding to the QDP produced by the target complex tone. This suggests that, for our stimuli at least, the FFR to the envelope of a complex tone can be measured without a major confounding effect of the QDP and without the need for a noise masker.

### Experiment 2B: Adaptor at 80 dB SPL

#### Rationale

Experiment 2A found no significant effect of the envelope rate of the complex-tone adaptor on the FFR strength for the target during period T-start, although there was a trend for the FFR to be somewhat lower when it was preceded by an adaptor with identical envelope rate (213 Hz) than when it was preceded by an adaptor with an envelope rate of 504 Hz. However, the FFR strength for the 504-Hz adaptor was quite weak and barely above baseline level (FFR strength in the 50-ms time range immediately before the presentation of the adaptor) for some subjects. In order to increase the amount of adaptation observed, and also to increase the FFR strength in response to the adaptors, experiment 2B repeated conditions AC213 and AC504 from experiment 2A with the level of the adaptor increased from 75 to 80 dB SPL.

#### Stimuli

The 213-Hz complex-tone target was always preceded by a 200-ms, 80-dB SPL complex-tone adaptor, i.e., the level of the adaptor was 5 dB above that of the target. The adaptor was a complex tone with an envelope rate of 213 or 504 Hz (conditions AC213 and AC504, respectively). We chose equal rms levels for the two adaptors as they were filtered into the same frequency region and, thus, would be equally affected by the middle ear transfer function.

Stimulus generation and presentation, and analysis of the recorded signal, were done in the same way as for experiment 2A, with the following exceptions: (i) Four blocks of 1500 trials were run for each condition, with conditions being alternated from one block to the next. (ii) The first and second time ranges for FFR analysis were from 12 to 62 ms after adaptor onset (A-start) and from 62 to 112 ms after adaptor onset (A-mid).

#### Subjects

Eleven subjects (four male) participated, two of whom also took part in experiment 2A. They ranged in age from 21 to 36 years and had self-reported normal hearing. The 11 were selected from a pool of 15 subjects because they showed clear FFR signals to the target alone and to the adaptors; at time A-start, their FFR was at least 8 dB (on average 19 dB) above baseline for both conditions. Four of them had some musical training. The experiment lasted about 3 h, including breaks.

#### Results

**FIG. 3:**
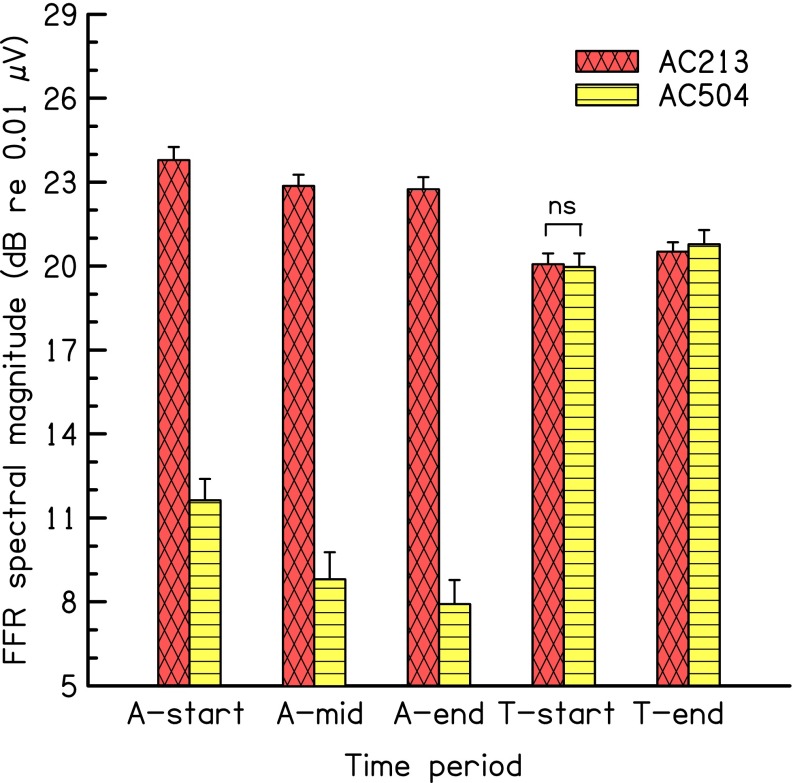
Results of experiment 2B. As in Figure 2, but for eleven subjects. Adaptor at 80 dB SPL, and target at 75 dB SPL; *ns* not significant.

Figure 3 shows the FFR strength averaged across subjects and the corresponding standard error across subjects. Note the increase in FFR strength during presentation of the adaptor relative to experiment 2A, especially for AC504, due to the increase of the adaptor level. Also note the lower FFR strength for the 213-Hz target than for the 213-Hz adaptor as a consequence of the 5-dB drop in level from adaptor to target and possibly further adaptation.

As in experiment 2A, adaptation over time is visible in the FFR in response to both adaptors. An RM two-way ANOVA (with factors time, A-start vs A-end, and adaptor type) was calculated on the FFR for both adaptors. The main effects of adaptor type and time were both highly significant [F (1,10) = 129.12, *p* < 0.001 and F (1,10) = 37.07, *p* < 0.001 for adaptor type and time, respectively]. There was also a significant interaction [F (1,10) = 16.39, *p* = 0.002], showing that there was significantly more adaptation (in dB) for the 504-Hz than for the 213-Hz adaptor. This is consistent with the results of experiment 1 that showed more adaptation for higher than for lower frequencies and F0s. Individual *t* tests, calculated for each adaptor type separately, showed that the FFR strength was significantly larger during period A-start than during period A-end for condition AC213 [difference =1.1 dB, t (10) = 4.69, *p* = 0.002] and condition AC504 [difference = 3.7 dB, t (10) = 5.40, *p* = 0.001].

To assess whether there was any evidence for envelope-rate-selective adaptation in the FFR for the target, a paired *t* test compared the FFR strength for the target during period T-start between the two adaptor conditions. The FFR strength in condition AC213 was not significantly smaller than for condition AC504 [difference = 0.1 dB, *p* = 0.85; two-tailed]. Thus, there is no evidence that the complex-tone adaptor with an envelope rate equal to that of the target reduced the FFR strength to the target more than the complex-tone adaptor with an envelope rate different from that of the target.

### Discussion

Like experiment 1, experiment 2B showed that FFR adaptation, measured in dB, was greater during presentation of a 504-Hz rate stimulus than during a lower-rate stimulus. Measured in this way, the results show an effect of modulation rate for stimuli filtered in the same frequency region and with similar excitation patterns. Thus, it seems that the effects of stimulus frequency and F0 on peristimulus adaptation are not exclusively driven by CF. Rather, F0 or envelope rate as such can affect the degree of adaptation in the peristimulus FFR. Such differences in amounts of adaptation might affect comparisons between FFR measures for stimuli with different F0s or envelope rates and duration.

We did not find any evidence that the envelope-related FFR in response to a complex-tone target is adapted (reduced) more by a complex-tone adaptor that has the same envelope rate as the target than by one with a different envelope rate. Hence, we found no evidence for tuning in the modulation domain in the FFR. Factors that would make it difficult to observe selectivity of adaptation with regard to envelope rate are: First, not all neurons in the IC and LL, and not all neurons that contribute to the FFR, have responses that are selective for envelope rate (Langner and Schreiner 1988; Joris et al. 2004). The responses of non-selective neurons would dilute the measured effects of any differences in adaptation produced by the three complex tone adaptors. Second, because the FFR requires averaging over several thousand trials, there is the possibility of accumulated adaptation effects across trials from the target itself. Specifically, stimuli were played with a repetition rate of 1.81/s, resulting in an inter-target interval (end of target to start of next target) of 452.5 ms. Possibly, envelope-rate-sensitive neurons firing in response to the target had not yet fully recovered from adaptation due to the previous target. This too would dilute the measured effect of any differential adaptation produced by the three complex-tone adaptors. Accumulated adaptation effects across trials could also occur from the adaptors, which were separated by an inter-adaptor interval of 352.5 ms. The observation of experiment 2A, that the FFR for AC213 at A-start was slightly smaller than that at T-start in condition AP213 suggests that recovery from adaptation produced by the adaptors was not complete at the beginning of a trial. However, this is unlikely to have reduced possible differences in adaptation between conditions, as the different adaptor conditions were tested in a blocked design (with breaks between blocks).

Interestingly, there is some recent evidence, from single-unit recordings in the IC of rabbits, that only a subset of units exhibit suppression in the discharge rate to a signal modulation when this is preceded by a masker modulation of the same rate (Wojtczak et al. 2011). Wojtczak et al. (2011) recorded from units that were sensitive to sinusoidal amplitude modulation (AM), and the units were stimulated at their CF with their best modulation frequency. The carrier was not interrupted between masker and signal modulation. Some units’ responses were even enhanced following the masker modulation. Overall, Wojtczak et al. (2011) reported only slightly lower firing rates when the signal modulation was preceded by a masker modulation of identical modulation rate than when it was preceded by an unmodulated carrier. If this observation of weak adaptation of neural responses to modulation per se holds in humans as well as rabbits, it might account for the present finding of equal target adaptation for all complex-tone adaptor rates in the FFR.

The results of experiment 2A showed that the envelope-related FFR in response to a complex-tone target showed a smaller reduction following a pure-tone adaptor with a frequency corresponding to the envelope rate of the target than following a complex-tone adaptor that was filtered into the same frequency region as the target complex. This provides strong evidence that the FFR response to the complex tone was not primarily driven by a propagated QDP and shows that an adaptor that is several octaves lower than the center frequency of the target did not produce any measurable adaptation. Carrier-frequency-specific adaptation in the FFR was investigated in experiment 3.

## **EXPERIMENT 3: SPECIFICITY OF ffr FOR AUDIO FREQUENCY**

### Rationale

Tuning in the audio frequency domain is well documented, and tonotopic maps have been reported throughout the auditory system starting from the cochlea to the auditory cortex (Palmer 1995). It has been observed psychophysically in both simultaneous and forward masking paradigms. Experiment 3 used adaptation to investigate whether and how well frequency selectivity is preserved in the FFR for low-frequency tones at moderate levels.

### Methods

#### Stimuli

The FFR was recorded for pure tones with frequencies of 213 or 504 Hz. To approximately equate the loudness of the two tones, the lower frequency tone (L) was presented at a level of 80 dB SPL and the higher frequency tone (H) at a level of 75 dB SPL (ISO 226 2003; Moore 2012). This was done to roughly equate the level at the cochlea, as the effect of frequency on threshold and loudness are mainly caused by the middle ear (Moore et al. 1997). Each tone functioned as either adaptor and/or target, leading to four main conditions: (i) adaptor of 213 Hz followed by target of 213 Hz (ALTL), (ii) adaptor of 504 Hz followed by target of 213 Hz (AHTL), (iii) adaptor of 213 Hz followed by target of 504 Hz (ALTH), and (iv) adaptor of 504 Hz followed by target of 504 Hz (AHTH). As in experiment 2, the adaptor duration was 200 ms while the target duration was 100 ms, including 5-ms raised-cosine rise/fall times.

When the adaptor and target had the same frequency (conditions ALTL and AHTH), the target phase was preserved relative to the adaptor phase; the adaptor and target comprised the first 200 and the last 100 ms of a 300-ms tone generated in sine starting phase, with onset and offset ramps applied to each. The starting phase of the target in condition AHTL was the same as that in ALTL, and the starting phase of the target in condition ALTH was the same as that in AHTH. In these four conditions, the adaptors always started in sine phase. An additional control condition (AH’TH) was run to check for any effect of starting phase of the adaptor relative to that of the target. In this condition, the adaptor started in cosine phase and the target had the same phase as in the other two conditions with the high-frequency target.

#### Electrophysiological Recording and Analysis

Stimulus generation and presentation, and analysis of the recorded signal, were done in the same way as in experiment 2, with two blocks of 1500 (valid) trials for each of the five conditions, and the 50-ms time ranges for FFR analysis set to 12–62 ms (A-start) and 62–112 ms (A-mid) for the first two time periods. Before analysis, the recorded waveforms for the two stimulus polarities were first subtracted from each other (to enhance phase locking information to the temporal fine structure) and the result divided by two, for each subject and condition. The FFR strength was defined as the highest magnitude present in the spectrum within a 20-Hz range centered at the frequency of the signal (adaptor or target), i.e., 213 or 504 Hz.

#### Subjects

Ten subjects (five male) participated, two of whom took part in experiment 2A, four of whom took part in experiment 2B, and two of whom took part in experiments 2A and 2B. They ranged in age from 19 to 37 years and had self-reported normal hearing. The 10 were selected from a pool of 15 subjects because they showed clear FFR signals to the adaptor signals (which also served as targets); at time A-start, their FFR was at least 7 dB (on average 21 dB) above baseline for all conditions. Three of them had some musical training. The experiment lasted about 3 h, including breaks.

### Results

**FIG. 4:**
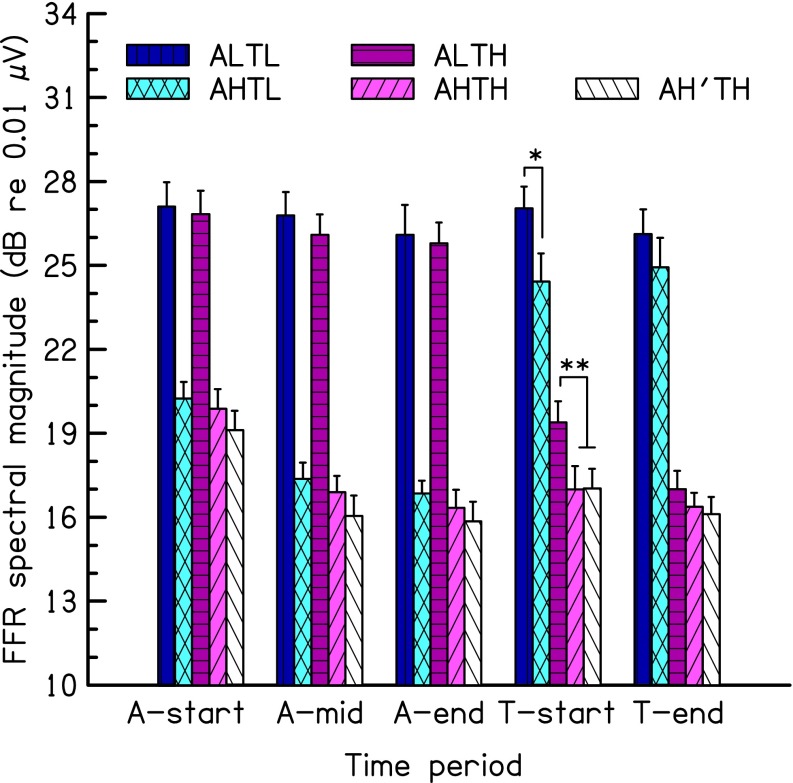
Results of experiment 3. Mean FFR spectral magnitude at the frequency of the pure-tone stimulus (adaptor or target) and the corresponding standard errors across 10 subjects. The three left-hand groups of five bars show the FFR strength during three time ranges of stimulation with the adaptor: 12–62 ms after onset (A-start), 62–112 ms after onset (A-mid), and 150–200 ms after onset (A-end). The two right-hand groups of five bars show the FFR strength during two time ranges of stimulation with the target: 12–62 ms after onset (T-start), and 50–100 ms after onset (T-end). Within each group of five bars, the two left-hand bars correspond to the conditions with the 213-Hz target, while the remaining three bars correspond to conditions with the 504-Hz target. Coding of conditions: *A* adaptor, *T* target, *L* low frequency (213 Hz) pure tone at 80 dB SPL, *H* high frequency (504-Hz) pure tone at 75 dB SPL; *p < 0.05; **p < 0.01.

Figure 4 shows the FFR strength averaged across the ten subjects and the corresponding standard errors. Adaptation over time is visible in the FFR in response to all adaptors (three groups of bars on the left-hand side). An RM two-way ANOVA (with factors time (A-start vs A-end), and condition (five levels)) was calculated on the FFR strength for all adaptors. The main effect of condition was highly significant [F (4,36) = 32.67, *p* < 0.001], indicating overall higher FFR strength for the low-frequency adaptor than for the high-frequency adaptor. The main effect of time was highly significant [F (1,9) = 44.7, *p* < 0.001]. Importantly, there was a significant interaction between condition and time [F (4,36) = 7.84, *p* = 0.006]. This interaction reflected the greater adaptation for the high-frequency than for the low-frequency adaptors, as observed in the previous experiments. The results of two RM two-way ANOVAs, one for the low-frequency adaptors and one for the high-frequency adaptors, showed that the effect of time was significant for both [F (1,9) = 36.45, *p* < 0.001 and F (1,9) = 5.92, *p* = 0.038, for the high-frequency and the low-frequency adaptors, respectively]; no other factor was significant. The mean reduction in FFR strength from period A-start to period A-end was 3.4 dB for the high-frequency adaptor and 1 dB for the low-frequency adaptor, values similar to those observed in the previous experiments.

If the FFR for the targets at T-start had been lower for an adaptor with an identical frequency than for an adaptor with a frequency different from that of the target, this would have provided evidence for frequency selectivity. However, this was not observed. Instead, for both target frequencies, the FFR to the target was lower when the target followed the high-frequency adaptor (Fig. 4, paler colored bars) than when it followed the low-frequency adaptor (darker colored bars); this difference did not depend on the target frequency. An RM two-way ANOVA (with factors target frequency, high vs low, and adaptor frequency, high vs low) was calculated on the FFR strength for the target during period T-start for all conditions with the adaptor starting in sine phase. As expected, the main effect of target frequency was highly significant [F (1,9) = 22.98, *p* = 0.001], indicating higher FFR strength for the low-frequency target than for the high-frequency target. The main effect of adaptor frequency was significant [F (1,9) = 20.01, *p* = 0.002], indicating more adaptation of the target following the high- than following the low-frequency adaptor. There was no significant interaction between target and adaptor frequency (*p* = 0.751). Thus, there is no evidence that the high-frequency adaptor reduces the FFR to the same (high) frequency target more than it reduces the FFR to the different (low) frequency target. The difference in FFR strength between conditions ALTH and AHTH (2.4 dB) was almost the same as the corresponding difference between ALTL and AHTL (2.6 dB). Thus, the larger effectiveness of the high-frequency adaptor was very similar for the low-frequency target and the high-frequency target. Therefore, the present results provide no evidence for frequency selectivity in the FFR.

Finally, there was no significant effect of the relative phase of the adaptor and target on the FFR to the target, at least for the 504-Hz tones; a paired *t* test comparing the FFR for period T-start between conditions AHTH and AH’TH showed no significant difference (*p* = 0.75).

### Discussion

In experiment 3, we found no evidence that the FFR in response to a pure-tone target is adapted (reduced) more by a pure-tone adaptor that has the same frequency as the target than by an adaptor with a different frequency. Instead, the high-frequency adaptor reduced the FFR to the target more than did the low-frequency adaptor, for both target frequencies. For *any* reduction in target FFR following an adaptor to occur, there must be at least some overlap between the neural populations excited by the adaptor and driving the FFR response to the target. Here, the difference between the FFR for a given target following the low- and high-frequency adaptors was just as great for the low-frequency target as it was for the high-frequency target. For this to occur, there must be a *substantial* overlap between the neural populations excited by the 504-Hz adaptor and driving the FFR response to the 213-Hz target.

The present observations can be compared with the results of a study investigating forward-masking patterns of the FFR in the cat. Snyder and Schreiner (1985) employed a 20-ms 800-Hz pure-tone probe and measured forward masking by 60-ms pure-tone maskers at various frequencies between 600 and 2400 Hz at various levels. From the masking functions, they determined a forward-masking tuning curve, showing the masker level necessary to produce a 30 % reduction in the FFR response to the probe as a function of masker frequency. They found that the tip of the V-shaped function, i.e., the best masking frequency, was one octave above the probe frequency and 24 dB below the probe level. They measured similar functions for the auditory nerve neurophonic (ANN). Snyder and Schreiner (1985) explained their findings by assuming that both the ANN and the FFR reflect vector summation of the phase-locked responses of fiber populations distributed along the basilar membrane. They noted that Kim et al. (1980) showed that the phase of the responses of auditory nerve fibers to a given tone changes steeply with CF for fibers with CFs below about twice the stimulus frequency and changes much more gradually for fibers with CFs higher than that. Thus, population-based measures of synchrony, like the ANN and FFR for medium to high-level stimuli, would mostly arise from fibers an octave or more above the stimulus frequency and therefore would be most efficiently masked by tones which are an octave above the probe frequency. Note, however, that Snyder and Schreiner (1985) implicated the AN as primary site of the origin for the ANN and for the FFR measured in the cat, as the effect of injections of drugs strongly suggested the origins for both to lie central to the generators of the cochlear microphonics and peripheral to the generators of waves II–V of the ABR. In contrast, the FFR measured in humans with the present electrode configuration is generally assumed to reflect sustained phase-locked neural activity mainly from rostral generators of the brainstem (IC and LL, Marsh et al. 1975; Smith et al. 1975; Glaser et al. 1976; Galbraith 1994; Krishnan 2006), which is consistent with the observed latencies of about 9 ms in the present studies. In spite of this difference in the presumed sites of generation, the present results are consistent with those of Snyder and Schreiner (1985), indicating that major characteristics of the human FFR may be “inherited” from population-based measures of synchronicity at the level of the AN.

We are aware of only one study of the effect of a preceding sound on the human FFR evoked by a target sound. Ananthanarayan and Durrant (1992) employed a 12-ms 500-Hz pure-tone probe and 60-ms pure-tone maskers at 0.5, 1, 2, and 4 kHz. Presumably, because of the short duration of the sounds they used, they referred to the measured effects as forward masking. However, their stimulus paradigm is similar to that used here and described here as adaptation. All tones had the same electric level (at the input to the headphones) as the 60-dB SL probe. The 0.5- and 1-kHz forward maskers reduced the FFR to the probe by about 3 and 14 dB, respectively, with no reduction for the higher-frequency maskers. The 3-dB reduction in the 500-Hz probe FFR following a same-frequency adaptor is similar to that observed in the present study (2.9 dB), despite the between-study differences. The 14-dB reduction in probe FFR following a 1-kHz adaptor is larger than that observed in the present study for an adaptor 1.24 octaves above the 213-Hz probe frequency. Further experiments are needed to determine the exact tuning of the human FFR to preceding maskers/adaptors.

The findings of both studies are broadly consistent with the results of a modelling study of Dau (2003). He suggested that the FFR for low-frequency, medium-to-high level tones represents synchronized activity mainly stemming from neurons with mid-to-high CFs, rather than from neurons with CFs around the signal frequency. He argued that the output of the basal region of the cochlear partition dominates the FFR because of the similar phase response of basal channels due to the high velocity of the traveling wave. Thus, according to Dau’s suggestion, the FFR observed for stimuli like the present ones originates from neurons on the upper skirts of the excitation patterns, which overlap substantially for different tone frequencies. As pointed out by Dau (2003), this interpretation of the FFR has important consequences for clinical applications, as it questions the role of the FFR as a neural correlate of low-frequency hearing. Nevertheless, at least for medium-level low-frequency pure tones, the FFR may be generated by activity along a restricted cochlear region which is however shifted toward the base relative to the probe frequency, as was concluded by Ananthanarayan and Durrant (1992).

As described in the “Introduction,” evidence from listeners with “dead” regions suggests that, when the temporal information is conveyed by neurons with CFs remote from the signal frequency, the pitch percept is weak and degraded (Huss and Moore 2005a, b). Therefore, it seems that, whatever the FFR measures, the neurons which dominate the measure are not the ones that normally convey the pitch of a sound. Thus, even for monaural stimuli, the FFR probably does not reflect pitch processing because it emanates from neurons that do not convey a clear pitch.

## **SUMMARY AND CONCLUSIONS**

The present study showed that:

- The FFR adapts more for stimuli with frequencies and F0s around 500 Hz than for lower frequencies and F0s. For pure tones at least, this effect did not depend on the overall magnitude of the FFR (experiment 1). Greater adaptation at high F0s was also observed for complex tones with unresolved harmonics filtered into the same frequency region but which differed in envelope rate, thus indicating an influence of stimulus rate per se rather than CF on this effect (experiment 2b). Such differences in amounts of adaptation might affect comparisons between FFR measures for stimuli of different frequencies, F0s, and envelope rates; when the stimuli are long, the difference between the FFRs for a low-rate stimulus and a high-rate stimulus might be larger than when the stimuli are shorter.
- The FFR for a harmonic complex tone filtered between 3.9 and 5.4 kHz with an envelope rate of 213 Hz was not reduced by a pure tone adaptor of 213 Hz (experiment 2A). This indicates that the FFR response to the complex tone was not substantially driven by a propagated distortion product with frequency equal to the envelope repetition rate. This method of checking for the effects of distortion products may be preferable to the use of a simultaneous noise masker, which can have undesired effects on the FFR over a wide frequency range (Gockel et al. 2012).
- The effect of a forward masker on the probe FFR was not tuned with regard to modulation rate. Thus, there was no evidence for tuning in the modulation domain in the FFR. Several possible reasons for this negative finding are discussed.
- In the audio-frequency domain, the FFR evoked by a 213-Hz probe was more reduced by a higher-frequency adaptor (504-Hz) than by an adaptor with the same frequency. This is consistent with models according to which the FFR for mid- to high-level tones originates mainly from neurons tuned to higher frequencies.
